# *Dusaceus* gen. nov., a new derbid genus of the tribe Cedusini (Hemiptera, Fulgoromorpha, Derbidae), with descriptions of two new species from China

**DOI:** 10.3897/zookeys.1280.186668

**Published:** 2026-05-29

**Authors:** Yong-Jin Sui, Zi-Zhong Li, Xiang-Sheng Chen, Lin Yang

**Affiliations:** 1 Institute of Entomology, Guizhou University, Guiyang, Guizhou, 550025, China Guizhou Key Laboratory of Agricultural Biosecurity, Guizhou University Guiyang China; 2 The Provincial Special Key Laboratory for Development and Utilization of Insect Resources of Guizhou, Guizhou University, Guiyang, Guizhou, 550025, China Institute of Entomology, Guizhou University Guiyang China; 3 Guizhou Key Laboratory of Agricultural Biosecurity, Guizhou University, Guiyang, Guizhou, 550025, China The Provincial Special Key Laboratory for Development and Utilization of Insect Resources of Guizhou, Guizhou University Guiyang China

**Keywords:** Breddiniolinae, new taxon, planthopper, taxonomy

## Abstract

A new planthopper genus, *Dusaceus* Sui, Li & Chen, **gen. nov**., is established within the tribe Cedusini Emeljanov, 1992 of the subfamily Breddiniolinae Fennah, 1950 (Hemiptera, Fulgoromorpha, Derbidae) to accommodate two new species from China: *D. lvchunensis* Sui, Li & Chen, **sp. nov**. (type species) and *D. suiyangensis* Sui, Li & Chen, **sp. nov**. Both species are described and illustrated in detail. A comprehensive checklist and an identification key to the genera of Cedusini from China are also provided.

## Introduction

The family Derbidae Spinola, 1839, with over 160 described genera and nearly 1700 species, is the third largest and most morphologically diverse family of planthoppers. The diversity of this family is significantly greater in tropical regions than in temperate zones (Bartlett et al. 2018; Emeljanov and Shcherbakov 2020; Dmitriev et al. 2022 onward; Bourgoin 2026). Derbidae is currently classified into three subfamilies and 22 tribes, two of which are extinct (Bourgoin 2026). Derbids are structurally diverse: the more derived tribes are readily distinguished by their distinctive morphology, whereas the basal ones often resemble Achilidae Stål, 1866 (Emeljanov and Shcherbakov 2020). A key diagnostic characteristic is that the terminal segment of the rostrum is substantially shorter than the subterminal segment, although this is not invariable (Bartlett et al. 2018). The higher classification of the Derbidae was most recently revised by Emeljanov (1995), who modified the system proposed by Fennah (1952) and reviewed the relevant morphology. This systematic framework was later updated by Banaszkiewicz and Szwedo (2005) and Szwedo (2005). The evolution of characters from basal tribes to more derived derbids was discussed by Emeljanov and Shcherbakov (2020).

The tribe Cedusini Emeljanov, 1992 was established by Emeljanov (1992). It is currently placed in the basal subfamily Breddiniolinae Fennah, 1950 (formerly known as Cedusinae Emeljanov, 1992). The subfamily Breddiniolinae was originally described as a tribe in the family Achilidae by Fennah (1950). It was later transferred to the family Derbidae based on male terminalia morphology (Emeljanov and Fletcher 2004). In a subsequent taxonomic revision, Emeljanov and Shcherbakov (2020) elevated Breddiniolini Fennah, 1950 to subfamily rank. Under the principle of priority, Cedusinae Emeljanov, 1992 is therefore treated as a junior subjective synonym of Breddiniolinae.

Cedusini is the largest tribe in the subfamily Breddiniolinae (Hemiptera, Derbidae), comprising nine genera (including one fossil genus) and approximately 245 described species (Bourgoin 2026). The genus *Cedusa* Fowler, 1904 is the most species-rich within the tribe, accounting for much of its diversity (Bartlett et al. 2018). To date, three genera and 13 species of Cedusini have been recorded in China (Yang and Wu 1994; Szwedo 2006; Sui et al. 2023).

In the present study, a new genus, *Dusaceus* Sui, Li & Chen, gen. nov., along with two new species, *D. lvchunensis* Sui, Li & Chen, sp. nov. and *D. suiyangensis* Sui, Li & Chen, sp. nov., are described and illustrated based on specimens collected in Yunnan and Guizhou provinces, China. Additionally, a comprehensive checklist and an identification key to genera of Cedusini from China are provided.

## Materials and methods

The morphological terminology follows Bourgoin (1987), Bourgoin and Huang (1990), and Yang and Wu (1994). Body length was measured from the apex of vertex to the tip of forewing using a Keyence VHX 1000E system. The standard terminology of venation follows Bourgoin et al. (2015). Descriptions and illustrations were based on dried specimens. External morphology was observed under a stereoscopic microscope (Nikon SMZ 1270), and all measurements were taking using an ocular micrometer. Colour images of adult habitus and male terminalia were captured with a Keyence VHX 6000 system. For genitalia examination, the genital segments of specimens were macerated in 10% NaOH and subsequently drawn from glycerine jelly preparations under a Leica MZ 12.5 stereomicroscope. The illustrations were scanned with a Canon CanoScan LiDE 220 scanner and further processed using Adobe Photoshop CS5 for labelling and figure composition. Dissected terminalia were preserved in glycerine within small plastic tubes, which were pinned alongside the corresponding specimens.

The type specimens and other examined specimens are deposited in the Institute of Entomology, Guizhou University, Guiyang, Guizhou Province, China (**GUGC**).

## Taxonomy

### Order Hemiptera Linnaeus, 1758

**Suborder Fulgoromorpha Evans, 1946**

**Superfamily Fulgoroidea Latreille, 1807**

**Family Derbidae Spinola, 1839**

**Subfamily Breddiniolinae Fennah, 1950**

#### Tribe Cedusini

Taxon classification

Animalia

HemipteraDerbidae

Emeljanov, 1992

82A66765-29CF-5A6B-9137-93D1A0EBF374

##### Type genus.

*Cedusa* Fowler, 1904.

##### Diagnostic characters.

Sensory pits absent on head and forewing. Second segment of rostrum longer than wide, its length less than twice its width. Subantennal process well developed. Forewing with the clavus closed; CuA branches non-anastomosing. Hindwing jugal margin lacking a stridulatory plate. In the female, abdominal sternite VII not fused with tergite VIII. Hind tibia without lateral spines. Hind leg spinal formula usually (5–9)-(5–6)-(4–6).

### Checklist of the genera and species of the tribe Cedusini from China

*Dusaceus* Sui, Li & Chen, gen. nov.

*D. lvchunensis* Sui, Li & Chen, sp. nov.

*D. suiyangensis* Sui, Li & Chen, sp. nov.

*Hauptenia* Szwedo, 2006

*H. beibengensis* Sui & Chen, 2023

*H. daliensis* Sui & Chen, 2023

*H. fellea* (Yang & Wu, 1994)

*H. glutinosa* (Yang & Wu, 1994)

*H. idonea* (Yang & Wu, 1994)

*H. jacula* (Yang & Wu, 1994)

*H. magnifica* (Yang & Wu, 1994)

*H. tripartita* Rahman, Kwon & Suh, 2012

*Muiredusa* Szwedo, 2006

*M. brunnea* (Muir, 1914)

*M. ignota* (Yang & Wu, 1994)

*M. littorea* (Yeh & Yang, 1994)

*Produsa* Szwedo, 2006

*P. concava* (Yang & Wu, 1994)

*P. cubica* (Yang & Wu, 1994)

### Key to the genera of the tribe Cedusini from China (male)

**Table:** 

1	Male pygofer with dorsocaudal angle produced into a process (Yang and Wu 1994: fig. 34E)	***Produsa* Szwedo, 2006**
–	Male pygofer with dorsocaudal angle not produced	**2**
2	Gonostylus short and stout, dorsal projection distal (Yang and Wu 1994: fig. 39E)	***Hauptenia* Szwedo, 2006**
–	Gonostylus relatively slender, dorsal projection basal	**3**
3	Gonostylus (Figs 3C–E, 7C–E) with a long, slender process arising from ventral margin near base	***Dusaceus* Sui, Li & Chen, gen. nov**.
–	Gonostylus lacking such a process	***Muiredusa* Szwedo, 2006**

### Descriptions

#### Genus Dusaceus

Taxon classification

Animalia

Hemiptera

Sui, Li & Chen gen. nov.

2EC6CEF0-F7C8-50EC-B223-E81764F8E0BD

https://zoobank.org/21EF929A-CEB6-486D-BB68-C208CB4809DB

##### Type species.

*Dusaceus lvchunensis* Sui, Li & Chen, sp. nov., here designated.

##### Description.

**Figure 1.**
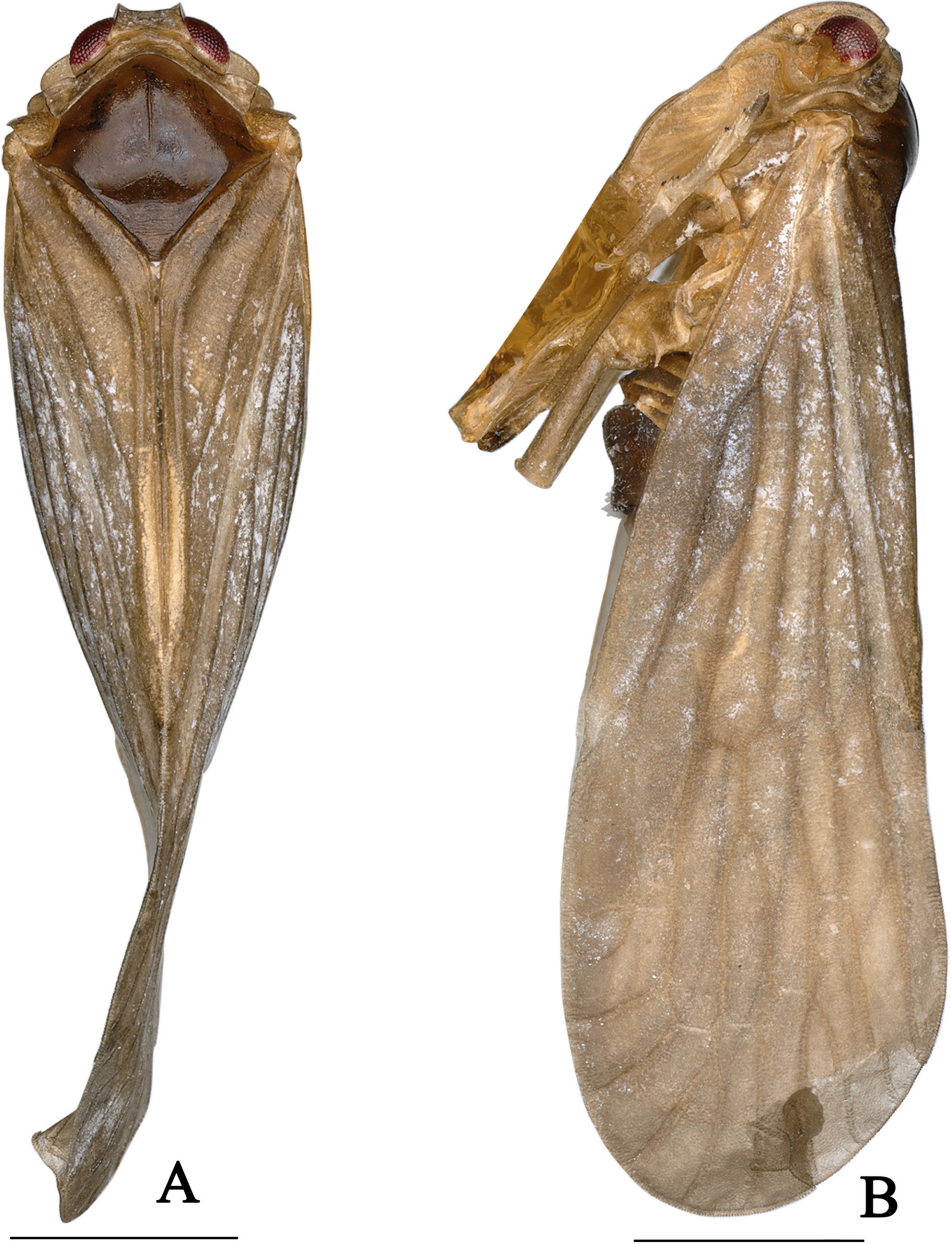
*Dusaceus lvchunensis* Sui, Li & Chen, sp. nov., male holotype. **A**. Habitus, dorsal view; **B**. Habitus, lateral view. Scale bars: 1 mm.

**Figure 2.**
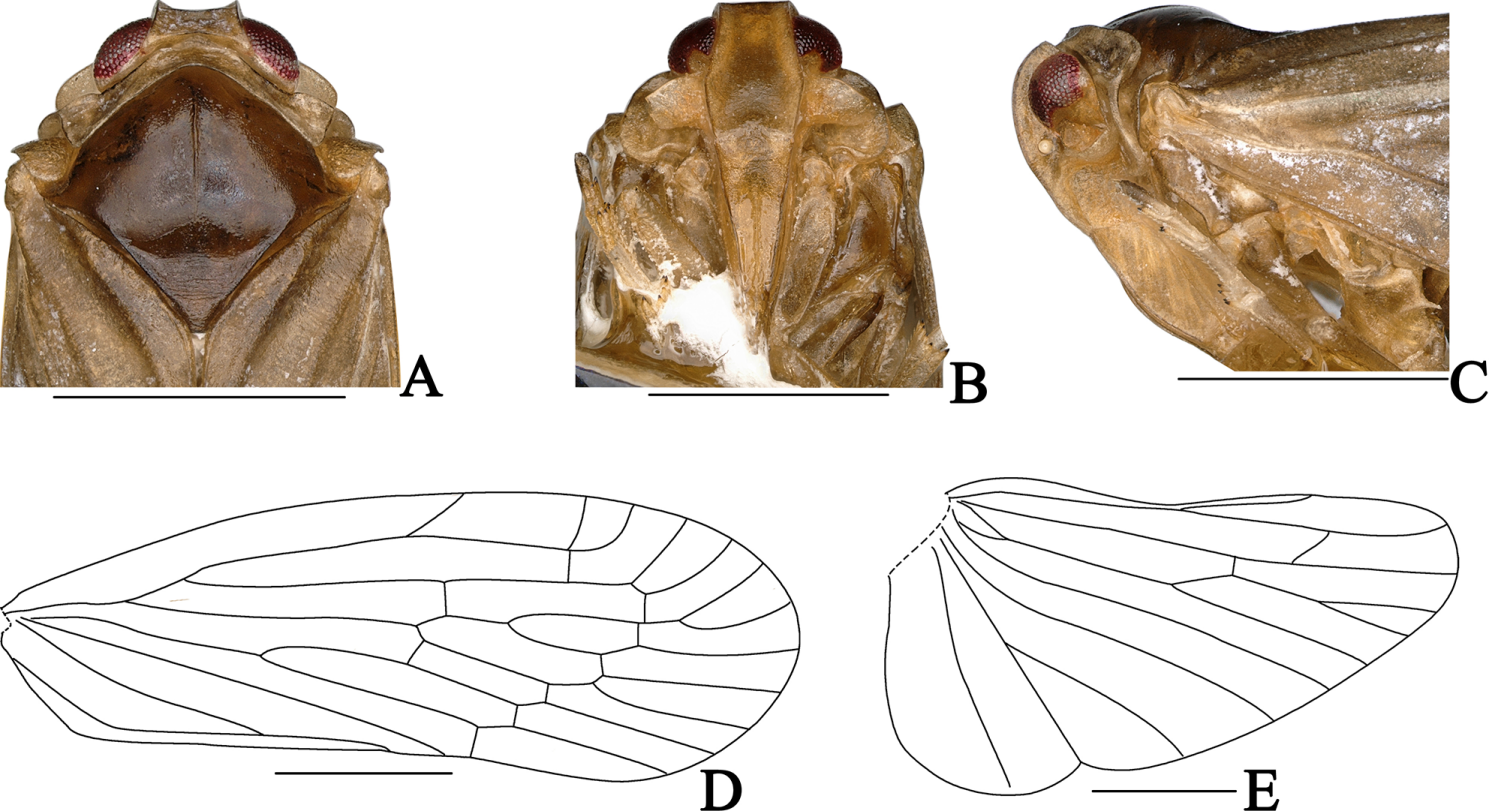
*Dusaceus lvchunensis* Sui, Li & Chen, sp. nov., male. **A**. Head and thorax, dorsal view; **B**. Face; **C**. Head and thorax, left lateral view; **D**. Forewing; **E**. Hindwing; **A**–**C**. Holotype; **D**, **E**. Paratype. Scale bars: 1 mm.

Head (Figs 1A, 2A) with eyes distinctly narrower than pronotum; vertex (1A, 2A) widely trapezoidal, with anterior margin (between lateral carinae of frons) two-thirds as wide as posterior margin; median carina absent; posterior margin slightly concave; lateral margins of frons anteriorly extending in dorsal view. Frons (Figs 2B, 6A) without median carina, concave, with lateral margins elevated and delimited from vertex by a distinct, transverse carina. Postclypeus (Figs 2B, 6A) longer at median line than frons; both medial and lateral carinae present. Apical segment of rostrum slightly longer than wide, extending beyond hind coxae. Lateral ocelli (Figs 1B, 2C) present, below ventral margin of eyes. Subantennal process (Figs 1B, 2C, 6A) well developed. Gena with a carina (Fig. 2C) between subantennal process and lateral carina of frons; carina obliquely oriented. Antennal pedicel (Figs 1B, 2C, 6A) subglobose, slightly longer than wide.

Pronotum (Figs 1A, 2A) relatively short, subequal to vertex in midlength. In dorsal view, anterior margin follows contours of head; posterior margin deeply concave. Lateral carinae and ventral margins lacking a subfoliaceous expansion to form cup-shaped antennal fovea. Mesonotum (Fig. 2A, C) convex, elevated above vertex in profile, broader than long at midline, with a distinct median carina. Forewing (Figs 2D, 6B, 6C) narrow; postcostal cell long; RA with one or two terminals; RP with two terminals; MP with four or five terminals, forked slightly beyond midlength; CuA forked at level of claval veins junction; crossvein *m-cu* situated between MP_3+4_ and CuA_1_; clavus closed, with apex slightly exceeding forewing midlength. Hindwing (Figs 2E, 6D) with vein ScP+RA long, running subparallel to RP, reaching anterior margin near apical third; MP single; CuA with three terminals. Hind leg lacking lateral spines, spinal formula 6-5-5.

**Figure 3.**
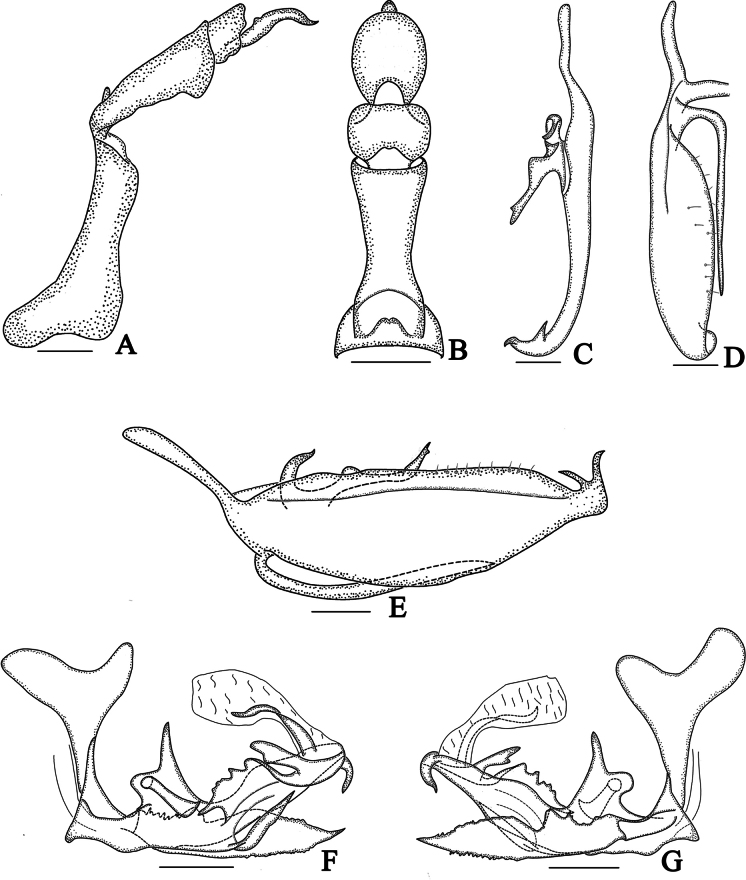
*Dusaceus lvchunensis* Sui, Li & Chen, sp. nov., male paratype. **A**. Pygofer and anal tube, left lateral view; **B**. Anal tube, dorsal view; **C**. Dorsal margin of right gonostylus, dorsal view; **D**. Ventral margin of right gonostylus, ventral view; **E**. Gonostylus, lateral view; **F**. Phallic complex, left lateral view; **G**. Phallic complex, right lateral view. Scale bars: 0.2 mm.

**Figure 4.**
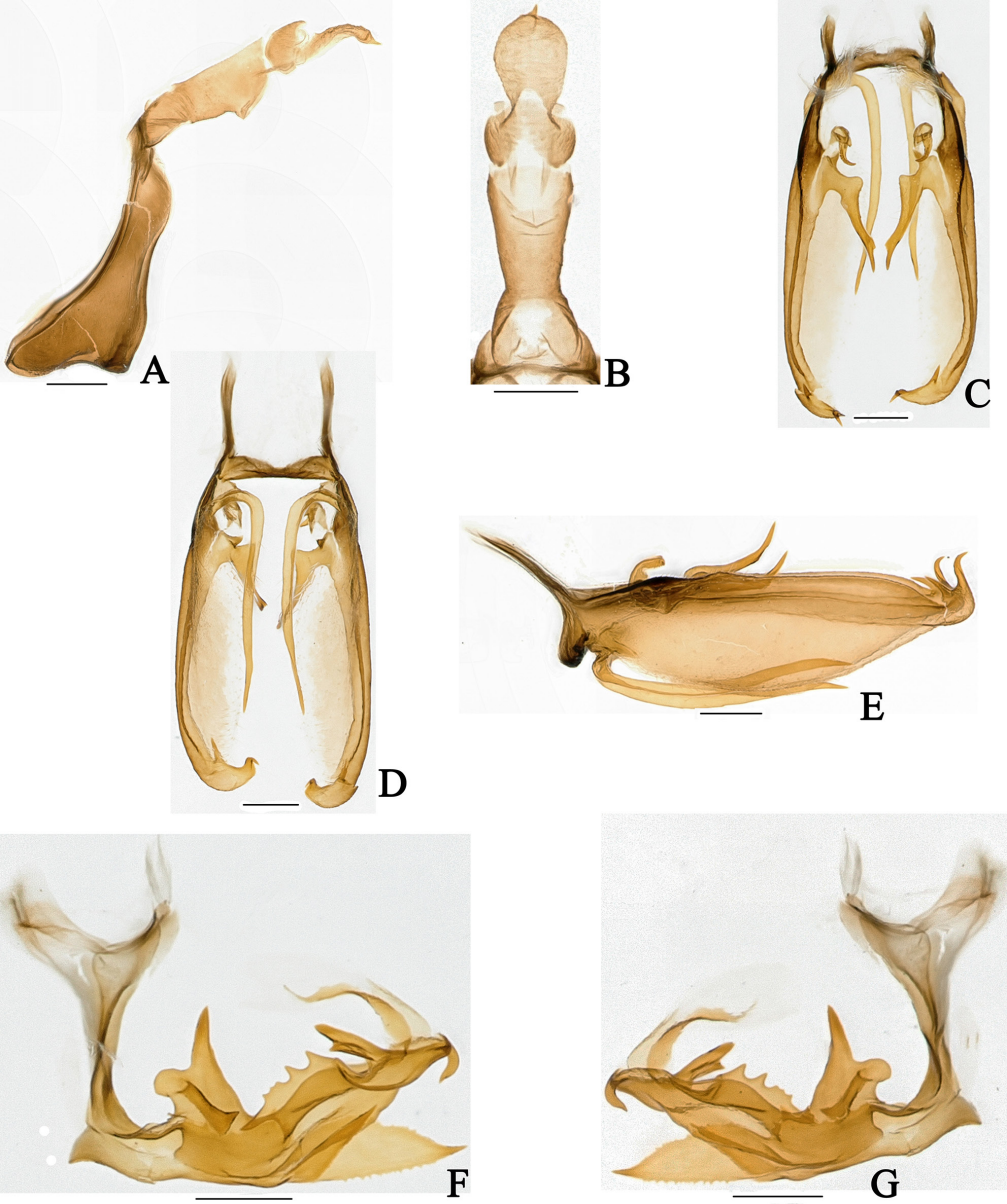
*Dusaceus lvchunensis* Sui, Li & Chen, sp. nov., male paratype. **A**. Pygofer and anal tube, left lateral view; **B**. Anal tube, dorsal view; **C**. Gonostyli, dorsal view; **D**. Gonostyli, ventral view; **E**. Gonostyli, lateral view; **F**. Phallic complex, left lateral view; **G**. Phallic complex, right lateral view. Scale bars: 0.2 mm.

Male pygofer (Figs 3A, 4A) in lateral view with dorsal margin distinctly shorter than ventral margin; dorsocaudal angle not produced. Male anal tube (Figs 3B, 4B) moderately long, dorsal margin longer than ventral margin in lateral view; segment XI (epiproct and paraproct) (Figs 3A, 4A, 7A, 8A) apically inserted. Gonostyli (Figs 3C–E, 4C–E, 7C–E, 8C–E) with basal dorsal projection; with a long, slender process arising from ventral margin near base, greatly exceeding middle of ventral margin.

##### Remarks.

The new genus is externally similar to other genera of Cedusini from China. It can be distinguished by the following combination of characters: male pygofer with dorsocaudal angle not produced (also not produced in *Hauptenia* and *Muiredusa*; produced into a process in *Produsa*); male anal tube with the dorsal margin longer than ventral margin (also longer in *Muiredusa*; shorter than ventral margin in *Hauptenia* and *Produsa*); gonostylus with dorsal projection basal (also basal in *Muiredusa* and *Produsa*; dorsal projection distal in *Hauptenia*); male paraproct not apically forked (also not forked in *Hauptenia* and *Produsa*; apically forked in *Muiredusa*); gonostylus with a long process on the ventral margin near its base (such a process lacking in *Hauptenia*, *Produsa*, and *Muiredusa*).

##### Etymology.

The generic name is an arbitrary combination of letters. Gender masculine.

##### Distribution.

China (Yunnan, Guizhou).

###### Key to the species of genus *Dusaceus* Sui, Li & Chen, gen. nov. (male)

**Table:** 

1	Gonostyli apically furcate (forked); periandrium with a broad, sheet-like process on the right side at base	***D. lvchunensis* Sui, Li & Chen, sp. nov**.
–	Gonostyli apically entire (not forked); periandrium with a long, slender, sheet-like process on the right side at base	***D. suiyangensis* Sui, Li & Chen, sp. nov**.

#### Dusaceus lvchunensis

Taxon classification

Animalia

Hemiptera

Sui, Li & Chen sp. nov.

828B4B3E-BB77-5E37-B74B-3F57F7CEE094

https://zoobank.org/B98F9ADC-78A0-46CD-9E5B-095B2908F016

Figs 1, 2, 3, 4, 5

##### Type material.

***Holotype*** ♂ (GUGC), China • Yunnan, Lvchun, 13 Aug. 2014, Y-J Wang (no. Der14081301). ***Paratypes***, • 1♂ (GUGC), same data as holotype (no. Der14081302); • 2♂♂ (GUGC), Z-X Zhou, other data same as holotype (no. Der14081303, Der14081304); • 2♂♂ (GUGC), Yunnan, Lvchun, 4 Aug. 2023, Y-J Sui (no. Der23080401, Der23080402).

**Figure 5.**
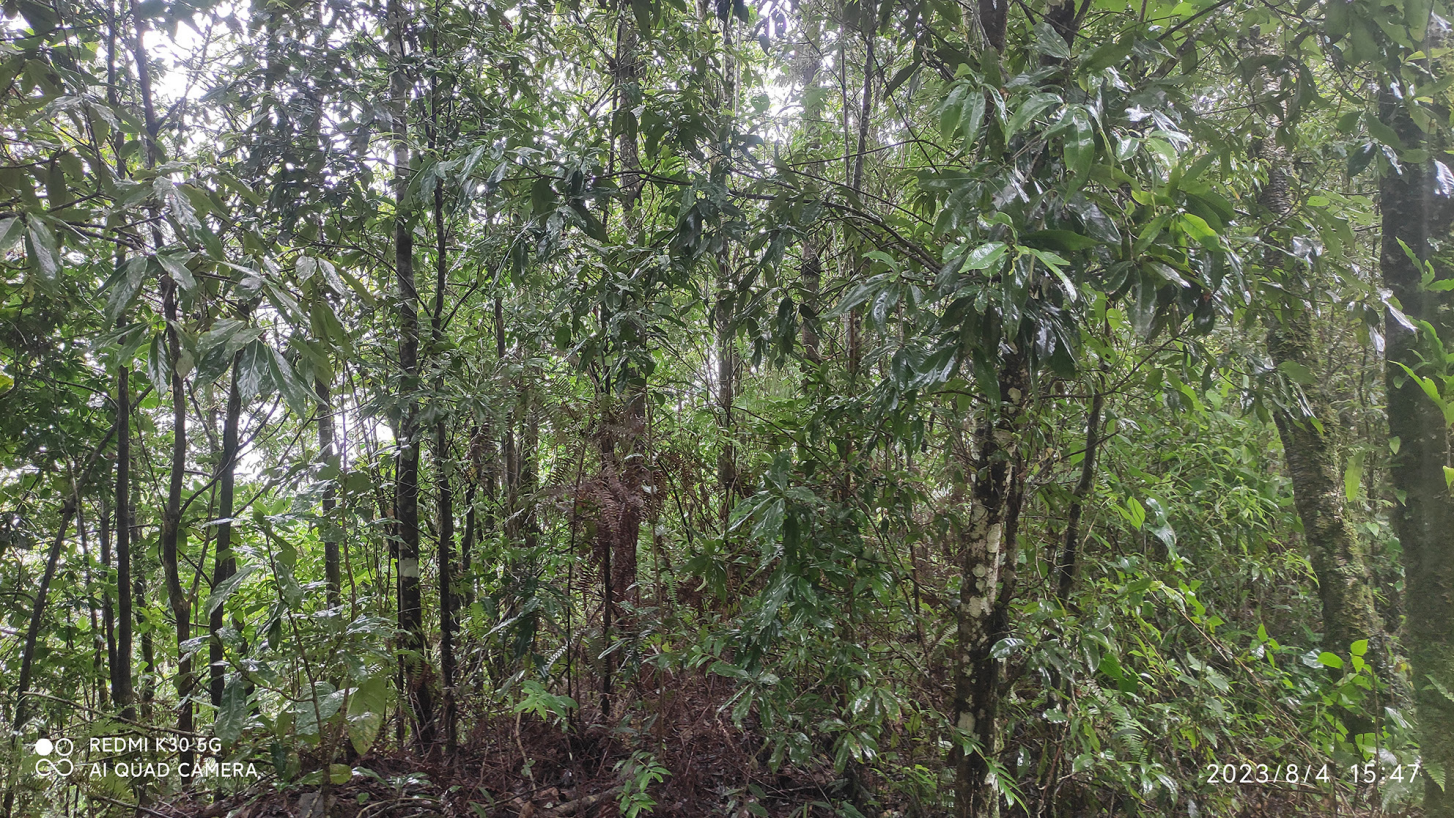
Habitat of *Dusaceus lvchunensis* Sui, Li & Chen, sp. nov. Two male paratype specimens collected from Lvchun County, Yunnan Province, China, on 4 August 2023.

##### Measurements.

Body length (including forewing): male 5.6–6.0 mm (*n* = 6); forewing length: male 5.1–5.3 mm (*n* = 6).

##### Description.

***Colouration***. General colour pale brown (Fig. 1). Vertex (Figs 1A, 2A) yellowish brown. Frons (Fig. 2B), clypeus, gena, antennae, and subantennal process dark yellow (Fig. 2C). Eyes (Figs 1, 2A–C) fusco-testaceous; lateral ocelli (Fig. 2C) yellowish white. Rostrum dark yellow, apex fuscous. Pronotum and tegula (Figs 1A, 2A) yellowish brown. Mesonotum (Figs 1A, 2A) brown. Forewing (Fig. 1) pale brown, veins concolourous. Hindwing subhyaline, greyish white; veins pale black. Thorax (Fig. 1B) with ventral areas yellowish brown. Legs (Fig. 1B) brownish yellow. Male terminalia dark brown.

***Head and thorax***. Head (Figs 1A, 2A) with eyes distinctly narrower than pronotum (ca 1:1.37), short. Vertex (Figs 1A, 2A) in dorsal view trapezoidal, ratio of width between basal angles to median length approximately 5.1:1, slightly projecting in front of eyes; apical margin distinctly carinate; posterior margin concave; lateral carinae elevated, median carina absent; disc slightly depressed. Frons (Fig. 2B) longer than wide (ratio of median length to maximum width approximately 1.42:1), shorter than clypeus (ratio approximately 1:1.45); disc depressed in entire length, lateral margins carinate. Postclypeus (Fig. 2B) with median and lateral carinae. Apical segment of rostrum slightly longer than wide. Antennae (Fig. 2C) short; pedicel subglobose; flagellum arising from apical point. Subantennal processes (Fig. 2C) distinct, ear-shaped. Gena with transversely oblique carina (Fig. 2C) between subantennal process and lateral carina of frons; transverse carina becoming indistinct near its end. Eyes (Fig. 2C) semicircular. Lateral ocelli (Fig. 2C) distinct, adjacent to both eyes and antennae. Pronotum (Fig. 2A) median length, subequal to that of vertex; anterior margin between eyes broadly convex; length behind eyes equal to median length; posterior margin deeply concave. Mesonotum (Fig. 2A, C) dorsally elevated, elevated above the plane of vertex in lateral view, median carina slightly elevated, posterior end triangularly depressed. Forewing (Fig. 2D) narrow, ratio of length to maximum width approximately 2.8:1; clavus closed; RA with two terminals; RP with two terminals; MP with five terminals. Hindwing (Fig. 2E) shorter than forewing; ScP+RA long, reaching anterior margin near apical third; RP reaching apical margin; MP single; CuA with three terminals. Spinal formula of hind leg 6-5-5.

***Male terminalia***. Pygofer (Figs 3A, 4A) with dorsal margin distinctly shorter than ventral margin in lateral view; dorsocaudal angle not produced. Anal tube (Figs 3B, 4B) moderately long. In lateral view (Figs 3A, 4A), dorsal margin longer than ventral margin, gradually widening from base to apex; segment XI (epiproct and paraproct) (Figs 3A, 4A) inserted apically; paraproct elongated. In dorsal view (Figs 3B, 4B), ratio of basal width to narrowest part approximately 2.37:1; ratio of median length (including epiproct and paraproct) to maximum basal width approximately 3.18:1. Gonostyli (Figs 3C–E, 4C–E) symmetrical or nearly so, apically furcate; with dorsal projection basal; a long, slender process arising from ventral margin near base, extending to near apical third of ventral margin. Phallic complex (Fig. 3F, G, 4F, G) asymmetrical. Periandrium robust, slightly curved. In left lateral view (Figs 3F, 4F), a large, irregular process arising from dorsal margin near base; its dorsoapical angle of process produced into a long, finger-shaped projection dorsally directed; a long lamina situated on left side of this irregular process near dorsal margin; a long, apically acute process arises from middle of ventral margin; a long, sheet-like process arising from middle to apex, its dorsal margin irregularly wavy. In right lateral view (Figs 3G, 4G), a large, broad, sheet-like process arising from base, exceeding apex of periandrium; apex of periandrium produced into a ventrally directed, hooked process visible in both lateral views. Endosoma (Fig. 3F, G, 4F, G) simple, with a long, torsional lamina on left side near middle, and a large membranous process on right side.

##### Remarks.

The new genus currently comprises only two species: *D. lvchunensis* sp. nov. (collected from Yunnan) and *D. suiyangensis* sp. nov. (collected from Guizhou). Although the sole known male specimen of *D. suiyangensis* was damaged during dissection, the two species can still be readily distinguished based on the male terminalia. The key differences are as follows: gonostyli apically furcate (apically not furcate in *D. suiyangensis*); gonostyli ventral process extending to near the apical third of its margin (extending nearly to the apex in *D. suiyangensis*); phallic complex similar in general structure, but shape of homologous processes markedly different (compare Figs 3F, 3G, 4F, 4Gwith 7F, G, 8F, G).

##### Etymology.

The specific epithet is derived from the type locality, Lvchun County, Yunnan Province, China.

##### Distribution.

Known only from the type locality.

#### Dusaceus suiyangensis

Taxon classification

Animalia

Hemiptera

Sui, Li & Chen sp. nov.

32A6DBF1-FD17-5A03-BBFF-F24AD3EAFD9A

https://zoobank.org/105E8098-629B-4B1C-839B-32D5D1CF17A8

Figs 6, 7, 8

##### Type material.

***Holotype*** ♂ (GUGC), China • Guizhou, Suiyang, 23 Aug. 2020, S-S Lv (no. Der20082301).

**Figure 6.**
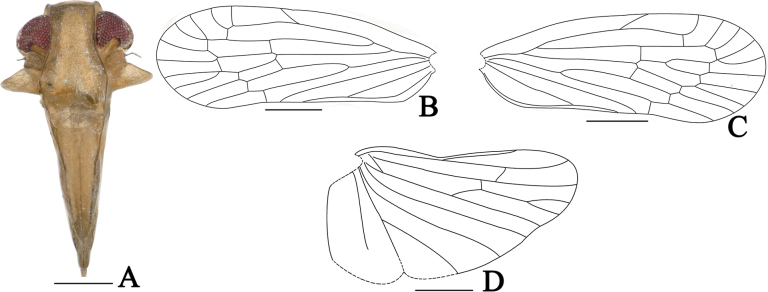
*Dusaceus suiyangensis* Sui, Li & Chen, sp. nov., male holotype. **A**. Face; **B**. Left forewing; **C**. Right forewing; **D**. Hindwing. Scale bars: 1 mm.

**Figure 7.**
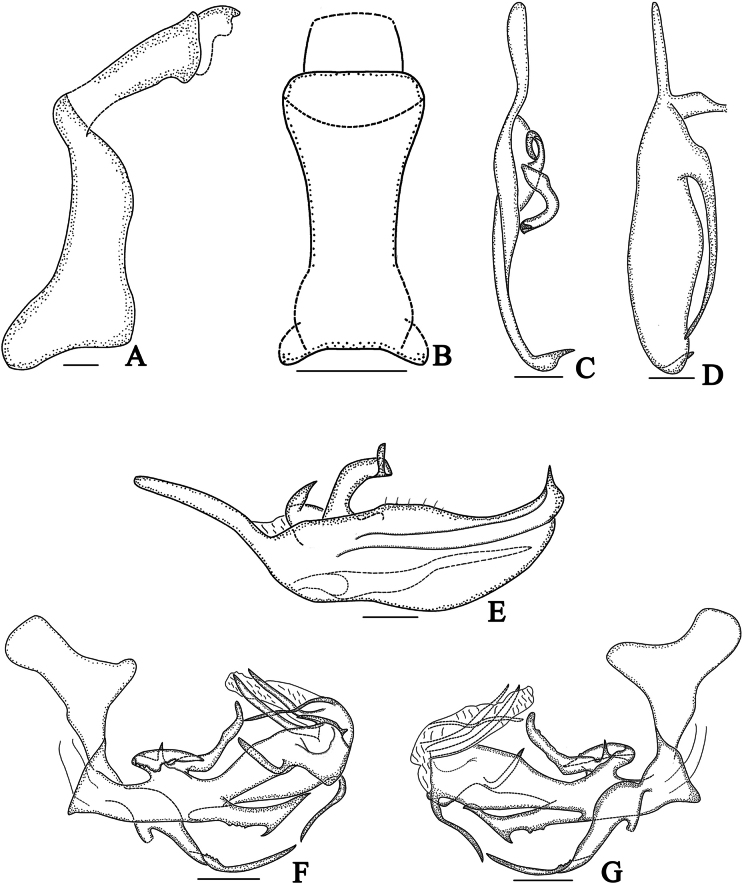
*Dusaceus suiyangensis* Sui, Li & Chen, sp. nov., male holotype. **A**. Anal tube and pygofer, left lateral view; **B**. Anal tube, dorsal view; **C**. Dorsal margin of left gonostylus, dorsal view; **D**. Ventral margin of right gonostylus, ventral view; **E**. Gonostylus, lateral view; **F**. Phallic complex, left lateral view; **G**. Phallic complex, right lateral view. Scale bars: 0.2 mm.

**Figure 8.**
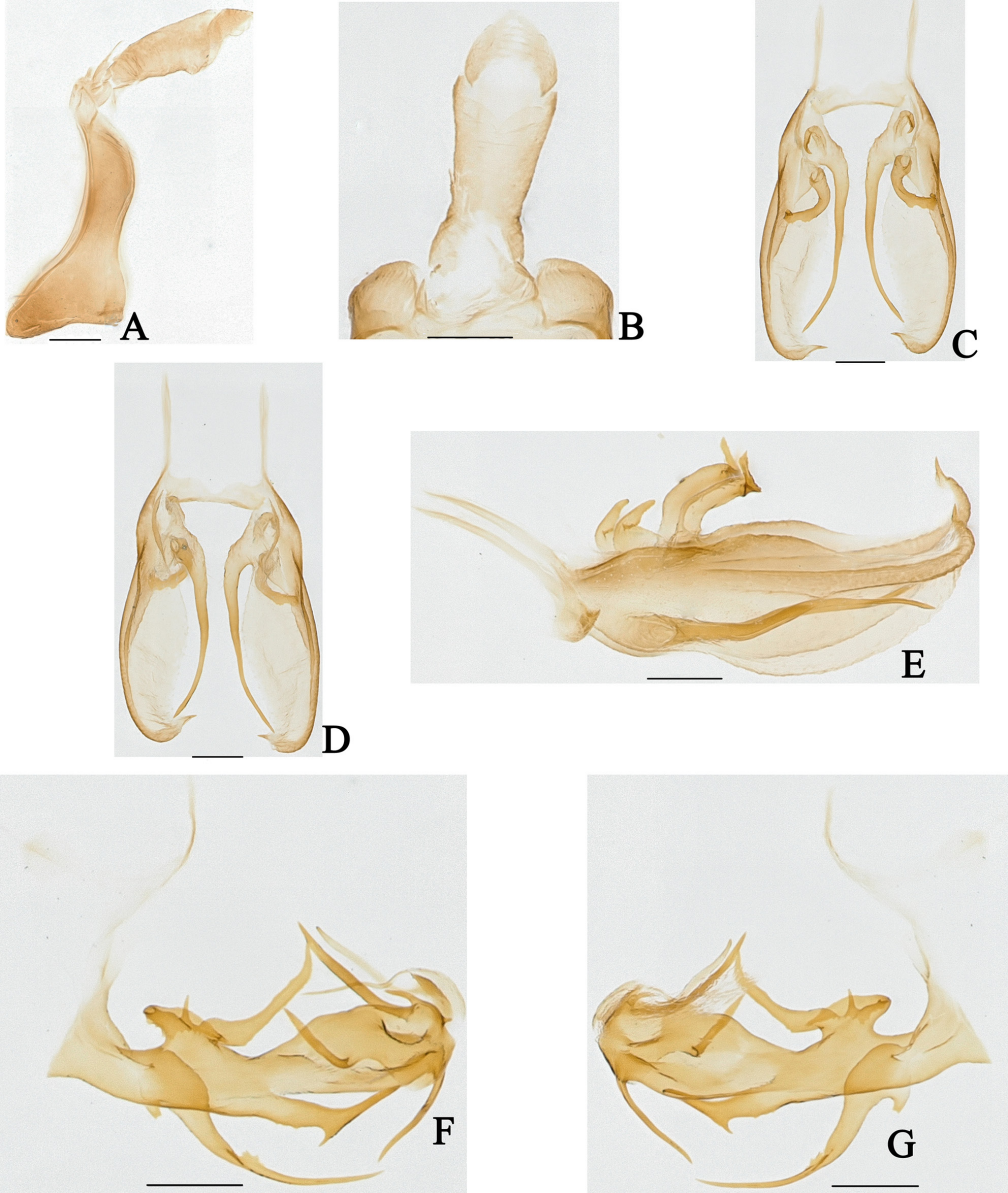
*Dusaceus suiyangensis* Sui, Li & Chen, sp. nov., male holotype. **A**. Anal tube and pygofer, left lateral view; **B**. Anal tube, dorsal view; **C**. Gonostyli, dorsal view; **D**. Gonostyli, ventral view; **E**. Gonostyli, lateral view; **F**. Phallic complex, left lateral view; **G**. Phallic complex, right lateral view. Scale bars: 0.2 mm.

##### Measurements.

Forewing length: male 4.96 mm (*n* = 1).

##### Description.

***Colouration***. Frons (Fig. 6A), clypeus, gena, antennae, and subantennal process dark yellow. Eyes fusco-testaceous; lateral ocelli yellowish white. Forewing pale brown; veins concolorous. Hindwing subhyaline, greyish white; veins pale black. Legs brownish yellow. Terminalia brown.

***Head and thorax***. Frons (Fig. 6A) longer than wide (ratio of median length to maximum width approximately 1.44:1), shorter than clypeus (ratio approximately 1:1.26); disc depressed over entire length; lateral margins carinate. Postclypeus (Fig. 6A) with median and lateral carinae. Antennae (Fig. 6A) short; pedicel subglobose; flagellum arising from apical point. Subantennal processes (Fig. 6A) distinct, ear-shaped. Gena with a transversely oblique carina between subantennal process and lateral carina of frons; this carina becomes indistinct near its end. Eyes semicircular. Lateral ocelli distinct, adjacent to both eyes and antennae. Forewing (Fig. 6B, C) narrow; ratio of length to maximum width approximately 2.7:1; clavus closed; RA with two terminals in left forewing (only one in right); RP with two terminals; MP with four terminals in left forewing (five in right). Hindwing (Fig. 6D) shorter than forewing; ScP+RA long, reaching anterior margin near apical third; RP reaching apical margin; MP single; CuA with three terminals. Spinal formula of hind leg 6-5-5.

***Male terminalia***. Pygofer (Figs 7A, 8A) in lateral view with dorsal margin distinctly shorter than ventral margin; dorsocaudal angle not produced. Anal tube (Figs 7B, 8B) moderately long. In lateral view (Figs 7A, 8A), dorsal margin longer than ventral margin, gradually widening from base to apex; segment XI (epiproct and paraproct) (Figs 7A, 8A) apically inserted. Gonostyli (Figs 7C–E, 8C–E) symmetrical or nearly so, apically acute, not furcate; with dorsal projection basal; a long, slender process arising from ventral margin near base, extending to near apex of ventral margin. Phallic complex (Figs 7F, 7G, 8F, 8G) asymmetrical. Periandrium robust, slightly curved. In left lateral view (Figs 7F, 8F), three processes arise from dorsal margin near base, with rightmost process longest; a strong, apically acute process arising from middle of ventral margin, bearing a sharp spine near its midlength; a slender, sheet-like process arising near apex. In right lateral view (Figs 7G, 8G), a long, slender, sheet-like process arises from the base, furcate near base, torsional near middle, apically acute; apex of periandrium produced into a long, slender, ventrally directed process, visible in both lateral views. Endosoma (Figs 7F, 7G, 8F, 8G) simple, with three long, slender processes and a large, subrectangular, membranous process.

##### Remarks.

Due to the damage to the sole specimen during dissection, some morphological details are incomplete. Nonetheless, based on the remaining structures and its general similarity to other species in the subtribe Cedusina Emeljanov, 1992, *D. suiyangensis* sp. nov. is considered very similar to *D. lvchunensis* sp. nov. The primary diagnostic differences lie in the structure of the male genitalia.

##### Etymology.

The specific epithet is derived from the type locality, Suiyang County, Guizhou Province, China.

##### Distribution.

Known only from the type locality.

## Discussion

The new genus is placed in the tribe Cedusini, subfamily Breddiniolinae. Cedusini is regarded as one of the early diverging lineages within Derbidae (Emeljanov 1995). Szwedo (2006) provided a key to the genera of this tribe; this key the genera were first divided into two groups based on structure of the forewing clavus, and three new genera (*Hauptenia*, *Muiredusa*, and *Produsa*) were established based on Chinese specimens. Emeljanov (2008) erected the subtribe Eocenchreina Emeljanov, 2008 for taxa possessing an achilid-like clavus; consequently, the three Chinese genera were indirectly placed in the subtribe Cedusina Emeljanov, 1992 (Dmitriev et al. 2022 onward; Bourgoin 2026). Thus, the tribe currently comprises two subtribes, Cedusina and Eocenchreina. These are primarily distinguished by clavus structure: Cedusina has a cixiid-like clavus, while Eocenchreina has an achilid-like clavus. Based on morphological characteristics, the new genus *Dusaceus* is assigned to the subtribe Cedusina.

**Figure 9.**
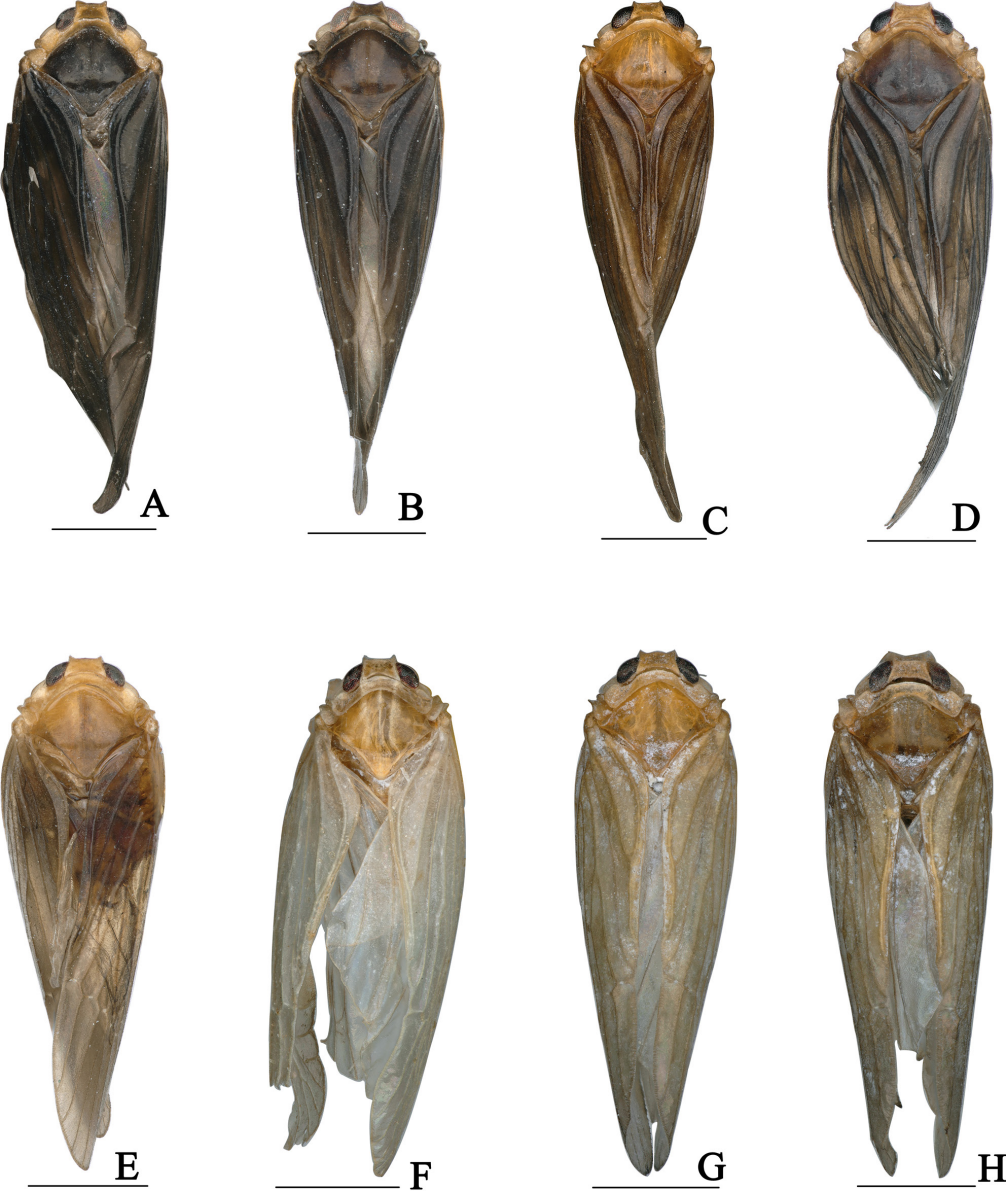
Some species of genus *Hauptenia* Szwedo, 2006. **A**. *H. beibengensis*; **B**. *H. daliensis*; **C**. *H. idonea*; **D**. *H. fellea*; **E**. *H. glutinosa*; **F**. *H. jacula*; **G**. *H. magnifica*; **H**. *H. tripartita*; **A**, **B**. Male holotypes; **C**–**H**. Male non-type. Scale bars: 1 mm.

During specimen examination, we found that the species of the Chinese subtribe Cedusina not only resemble each other closely within a genus (Fig. 9), but they also show little overall difference in appearance between genera. Only a few foreign species of subtribe Cedusina have published images of their habitus available for comparison. For example, Bahder et al. (2025) provided habitus and male genitalia figures of *Cedusa hampora* and *Cedusa yowza*, and Gnanapragasam et al. (2025) provided ecological photographs and partial male genitalia figures of *Cedusa vulgaris*. Compared with these species, their overall appearance is likewise similar, and the differences are almost entirely found in the male genital structures. While checking the literature, we also noticed that although this subtribe contains many species, the descriptions for a number of them are incomplete or lacking in detail, which has caused considerable difficulties for systematic classification. Apart from the new genus established here, we still have some specimens that cannot easily be placed in any existing genus. Based on the current situation, the most promising way to advance the morphological classification is probably to systematically collect fresh specimens and obtain corresponding molecular data, which can be used to support the current morphological taxonomy or to provide evidence for future morphological revision.

## Supplementary Material

XML Treatment for Cedusini

XML Treatment for Dusaceus

XML Treatment for Dusaceus lvchunensis

XML Treatment for Dusaceus suiyangensis

## Additional information

### Artificial Intelligence (AI) use

The authors accept full responsibility for the content of the manuscript, including the disclosure of any use of AI.

No AI tools were used in the preparation of this manuscript.

### Author ORCIDs

Y.-J. Sui https://orcid.org/0000-0001-7514-2984

Z.-Z. Li https://orcid.org/0000-0003-1609-9992

X.-S. Chen https://orcid.org/0000-0001-9801-0343

L. Yang https://orcid.org/0000-0002-7841-5156

### Data availability

All of the data that support the findings of this study are available in the main text.
